# Skin lesions suspected of malignancy: an increasing burden on general practice

**DOI:** 10.1186/1471-2296-15-29

**Published:** 2014-02-12

**Authors:** Cecile JL Koelink, Boudewijn J Kollen, Feikje Groenhof, Klaas van der Meer, Wouter K van der Heide

**Affiliations:** 1 Department of General Practice, University of Groningen, University Medical Center Groningen, PO Box 196, Huispostcode FA 20, 9700 AD Groningen, The Netherlands

**Keywords:** Skin Neoplasms, Family practice, Physicians, Family, Netherlands, Referral and consultation, Surgical procedures, Minor

## Abstract

**Background:**

Skin cancer is believed to impose a heavy burden on healthcare services, but the burden of skin lesions suspected of malignancy on primary healthcare has never been evaluated. Therefore the aim of this study was to determine the demand for care in general practice due to these suspected skin lesions (i.e. lesions that are suspected of malignancy by either the patient or the GP).

**Methods:**

Registry study based on data (2001–2010) from the Registration Network Groningen. This is a general practice registration network in the northern part of the Netherlands with an average annual population of approximately 30,000 patients. All patient contacts are coded according to the International Classification of Primary Care (ICPC). Consultations for skin lesions suspected of malignancy were selected according to the assigned ICPC codes. Subsequently, the number of consultations per year and the annual percent change in number of contacts (using the JoinPoint regression program) were calculated and analysed. Additionally, the percentage of patients referred to secondary care or receiving minor surgery within one year after the first contact were calculated.

**Results:**

From 2001 onwards we found an annual increase in demand for care due to skin lesions suspected of malignancy of 7.3% (p < 0.01) and in 2010 the benign:malignant ratio was 10:1. In total 13.0% of the patients were referred and after 2006, minor surgery was performed on 31.2% of the patients. Most surgeries and referrals took place within 30 days.

**Conclusions:**

Suspected skin lesions impose an increasing burden on primary healthcare and most likely on healthcare costs as well. General practitioners should therefore be trained in diagnosing skin lesions suspected of malignancy, as a high diagnostic accuracy can save lives in the case of melanoma, and may also prevent unnecessary, costly, excisions and referrals to secondary healthcare.

## Background

Skin cancer incidence is rising [1-6]. In the Netherlands, one in six people are expected to develop skin cancer [7]. Public awareness is also rising as a result of many public information campaigns [8-11] and this may lead to an increased consultation rate. These consultations also include non-malignant skin lesions. In fact, the majority of patients visiting their physician for a skin lesion suspected of malignancy do not have skin cancer. De Vries et al.has suggested that for every new case of skin cancer another 20–50 patients will consult their general practitioner (GP) or dermatologist [12]. This estimate lacks solid evidence, but seems to be in line with daily practice.

In the Netherlands, the GP has a gatekeeper role and patients visit their GP first for any health-related question. The GP can perform a diagnostic procedure, which may include an excision or referral to the dermatologist in the case of suspected lesions. Despite the large number of encounters for skin lesions, many GPs lack a solid training in dermatology [13,14]. In contrast to the UK [14], no specific guideline for skin lesions suspected of malignancy is available in the Netherlands.

We believe that knowledge on healthcare demands for skin lesions suspected of malignancy (i.e. lesions that are suspected of malignancy by either the patient or the GP) is important. It may identify areas for training as well as revealing possibilities for substitution of care.

Therefore, the aim of this study was to determine the demand for care in general practice due to skin lesions suspected of malignancy for the period 2001–2010. We were particularly interested in the consultation rates and subsequent treatments by GPs, including watchful waiting, excision of the lesion and referral to secondary care.

## Methods

We performed a retrospective analysis on data from the Registration Network Groningen (RNG). This network was established in 1989 and consists of patient registrations of three general practices with 17 GPs in the north-eastern part of the Netherlands. The RNG includes a dynamic population with an average annual population of approximately 30,000 patients. For all patients, both symptoms and diagnoses are coded (by the GPs), according to the International Classification of Primary Care (ICPC) [15,16]. Treatments such as minor surgery and referrals are registered as well. All GPs in this network are especially trained for this type of ICPC registration.

All patients aged 18 years and older were selected, with a consultation for skin lesions suspected of malignancy between 2001 and 2010. To identify consultations for skin lesion suspected of malignancy without running the risk of also selecting too many consultations for other reasons, 2 GPs (KvdM and WvdH; both > 25 years of experience) and 1 researcher (CK) selected the ICPC codes. Consequently, the following ICPC codes S26 (Fear of cancer of skin), S77 (Malignant neoplasm of skin), S79 (Benign neoplasm of skin, other), S80 (Unspecified neoplasm of skin, other), S81 (Haemangioma/lymphangioma), S82 (Naevus/mole), S83 (Congenital skin anomaly, other) and S99 (Skin disease, other) were used for this analysis. The latter ICPC code was included because it also includes verruca seborrhoica, kerato-acanthoma and actinic keratosis (see Additional file 1).

We calculated the annual number of contacts, referrals and minor surgery for lesions suspected of malignancy per 1,000 patients. For this, we first calculated the total number of patients per year in the database. As the RNG consists of a dynamic population, this was done by counting the true number of person-years for each year based on the actual days during the year in which the patient was present in the database (i.e. registered at one of the practices).

Subsequently, we also assessed which percentage of the patients received an intervention, i.e. either minor surgery performed by their GP or referral to secondary care, within 1 year after their first consultation for one of the above mentioned ICPC codes. Due to the small number of annual consultations (<25), codes S26, S81 and S83 were not included in the analysis. For minor surgery, we selected patients with a first visit from 2006 onwards. This period was chosen, because from that year onwards a new financial contract for GPs was introduced which led to improved registration of minor surgery. Before 2006 no trustworthy data on minor surgery could be retrieved. However, referrals have always been registered and for this intervention we selected the period from 2001 onwards. A first visit was defined as having no earlier contact for the analysed ICPC code in the database.

### Analyses

Slope differences from zero at alpha 0.05 and the annual percent change (APC) in number of contacts, referrals and minor surgery were estimated and analysed for trend significance, using the JoinPoint Regression Program, version 3.5.2. October 2011 of the Statistical Research and Applications branch of the US National Cancer Institute. This was done for all lesions as well as separately for malignant and benign lesions.

Descriptive analyses were used to report the percentage of patients being subjected to an intervention, the median time to the intervention and the percentage of patients with an intervention 30 and 90 days after the first consultation. For this, SPSS version 18 was used.

A difference with a p-value <0.05 was considered significant.

As all data were received anonymously no ethical approval for this study was needed. This was confirmed by the Medical Ethical Board of the University Medical Center Groningen.

## Results

On average, there were 22,343 patients aged 18 years and older per year in this study.

### Number of contacts per year

**Figure 1:**
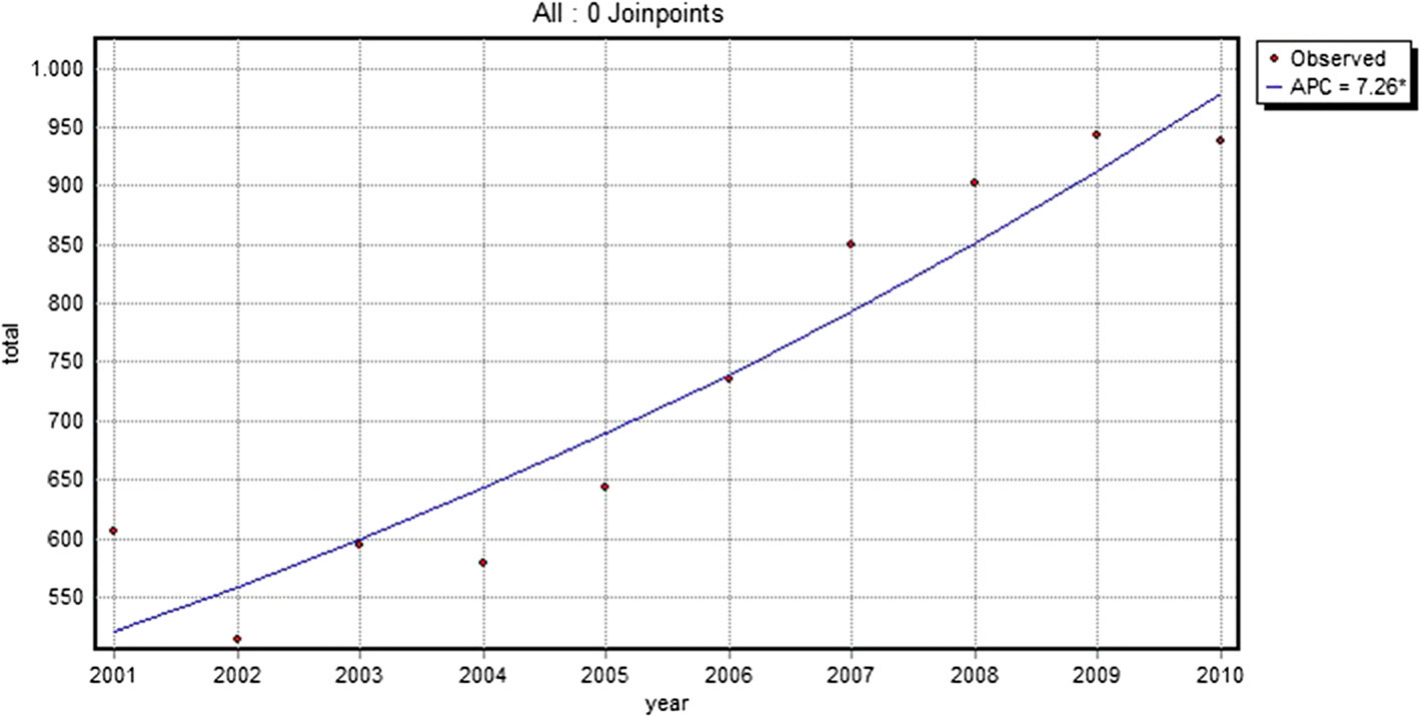
**Total number of contacts per 1,000 patients per year.** (line = trend line, APC = annual percent change).

**Figure 2:**
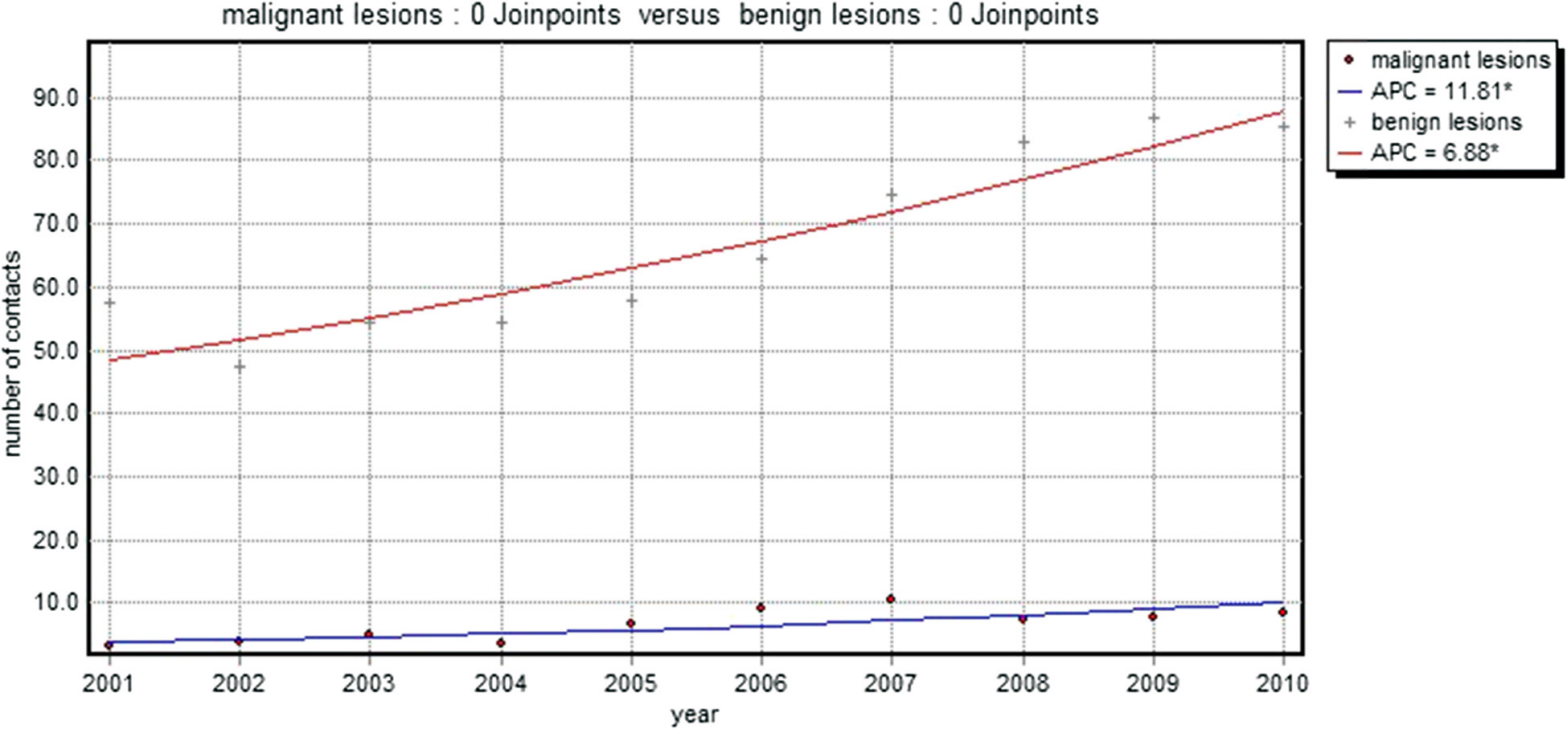
**Total number of contacts for malignant (•) and benign (+) skin lesions per 1,000 patients per year.** (line = trend line, APC = annual percent change).

From 2001 to 2010, 16,337 contacts of 7034 different patients (median number of contacts: 2) for skin lesions suspected of malignancy were registered. The total number of contacts per year increased by 54.8% from 60.6 contacts/1,000 patients in 2001 to 93.8 contacts/1,000 patients in 2010. This was a significant increase with an annual percent increase of 7.3 (p < 0.01) (Table 1, Figure 1). This increase was shown for both malignant (ICPC S77; annual percent change 11.8) and non-malignant (other ICPC codes; annual percent change 6.9) lesions (Figure 2). In 2010 only 1 in 10 skin lesions suspected of malignancy was malignant (Table 2).

**Table 1:** Number of contacts per year / 1,000 patients (raw number of consultations in the database)

	**S77 (Malignant neoplasm of skin)**	**S79 (Benign neoplasm of skin, other)**	**S80 (Unspecified neoplasm of skin, other)**	**S82 (Naevus/mole)**	**S99 (Skin disease, other)**	**S26; S81; S83 (Fear of cancer of skin, Haemangioma/lymphangioma and Congenital skin anomaly, other)**	**Total**
2001	3.3 (73)	20.9 (465)	3.6 (81)	22.9 (510)	8.7 (194)	1.1 (25)	60.6
2002	4.0 (88)	19.4 (427)	2.5 (55)	19.3 (425)	5.0 (110)	1.1 (24)	51.4
2003	4.8 (107)	19.8 (438)	1.7 (38)	22.1 (488)	9.5 (211)	1.4 (31)	59.4
2004	3.6 (81)	20.5 (461)	1.9 (42)	20.1 (452)	11.1 (249)	0.7 (15)	57.9
2005	6.7 (153)	22.7 (520)	0.9 (21)	19.9 (456)	13.4 (307)	0.7 (17)	64.4
2006	9.1 (199)	26.1 (574)	2.2 (48)	20.6 (452)	14.2 (311)	1.4 (31)	73.6
2007	10.4 (232)	24.5 (547)	4.0 (89)	28.1 (626)	16.7 (372)	1.2 (27)	85.0
2008	7.2 (161)	29.7 (667)	4.8 (109)	30.5 (685)	16.5 (372)	1.6 (36)	90.3
2009	7.6 (172)	26.2 (592)	5.4 (121)	33.0 (745)	20.8 (470)	1.2 (27)	94.3
2010	8.4 (189)	27.6 (621)	5.2 (116)	29.2 (657)	22.4 (504)	0.9 (21)	93.8

**Table 2:** Benign/malignant ratio of skin lesions suspected of malignancy per year

**Year**	**Benign: Malignant**
2001	17.5 : 1
2002	11.8 : 1
2003	11.3 : 1
2004	15.0 : 1
2005	8.6 : 1
2006	7.1 : 1
2007	7.2 : 1
2008	11.6 : 1
2009	11.4 : 1
2010	10.2 : 1

### Patients receiving minor surgery

**Figure 3:**
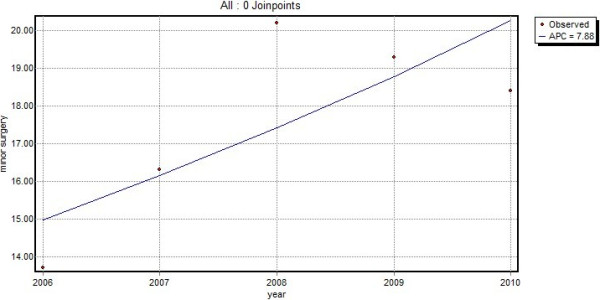
Minor surgery (number of excisions) per 1,000 patients per year (line = trend line, APC = annual percent change).

A total of 4,513 patients had a first visit for a skin lesion suspected of malignancy from 2006 onwards. In 31.2% of these patients, GPs performed minor surgery within one year after their first contact. The median time from the first contact to minor surgery was 6 days. After 30 days, 91.8% of all minor surgery had taken place, after 90 days this percentage increased up to 96.9%. The total number of patients receiving minor surgery increased from 13.7/1,000 patients in 2006 to 18.4/1,000 patients in 2010, which represents an annual percent increase of 7.9 (p = 0.13) (Figure 3).

### Patients referred to secondary care

**Figure 4:**
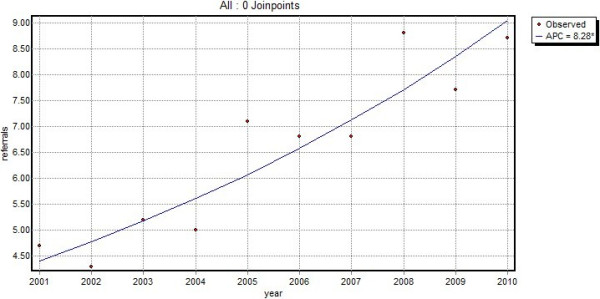
Total number of referrals per 1,000 patients per year (line = trend line, APC = annual percent change).

Of the 8,228 patients with a first contact for a skin lesion suspected of malignancy from 2001 onwards, 13.0% were referred to secondary healthcare at or within one year after the first consultation. As more than half of the patients were referred on the day of the consultation, the median time to referral was 0 days; 88.1% of the patients were referred within 30 days after the first visit and after 90 days, 92% of the referrals had taken place. The total number of referrals increased from 4.7/1,000 patients in 2001 to 8.7/1,000 patients in 2010. This corresponded to a significant annual increase of 8.3% (p < 0.01) (Figure 4).

## Discussion

### Summary of main findings

This study shows that skin lesions suspected of malignancy impose an increasing burden on general practice. During the period 2001–2010, the demand on care for skin lesions suspected of malignancy increased significantly (p < 0.01) with an annual percent increase of 7.3, leading to 93 contacts/1,000 patients/year in 2010. The majority of these contacts are due to benign lesions. A large proportion, 13.0%, of the new lesions are referred to secondary care and the GP performed minor surgery on more than 31% of the new lesions. Almost all referrals and minor surgery took place within 30 days after the first visit, suggesting that GPs make prompt decisions concerning the treatment.

### Context with other literature

In this study we found that a Dutch GP was consulted 93.8 times per year per 1000 patients older than 18 years in 2010. This increasing demand, if persevering, may eventually approach the estimated increase reported by de Vries et al. of 2 consultations/day in the year 2015 [12]. It is common knowledge that dermatology is a specialist area GPs often refer to [17]. This study shows that skin lesions suspected of malignancy must constitute a large group of these referrals. Within 1 year after the first visit, 13.0% of the patients were referred to secondary care. An even greater proportion of the patients, i.e. 31.2%, had their skin lesion removed by the GP. These percentages are comparable to the 10.2% and 27.4% respectively observed by van Dijk et al. [18] The minor differences observed are probably due to a different selection of skin lesions as van Dijk et al. only studied benign neoplasms of the skin and naevi, and included episodes of care instead of first contacts.

### Implications of results

As demonstrated by our study, GPs are frequently and increasingly confronted with the care of skin lesions suspected of malignancy. Most of these lesions are benign and although the number of contacts for malignant lesions are increasing at a higher rate than the number of contacts for benign lesions, in 2010, only 1 in 10 skin lesion suspected of malignancy was malignant. Furthermore, the total number of excisions (registered as minor surgery) and referrals showed an annual increase of 7.9% and 8.3% respectively, which is slightly higher than the 7.3% increase in the total number of contacts for skin lesions suspected of malignancy found in this study. This increasing demand for care in general practice, but as seen by the increase in referrals to secondary care as well, is likely to result in an increasing burden on healthcare costs. It is, therefore, important that GPs are adequately trained to diagnose and treat skin lesions that are suspected of malignancy as early detection can save lives, in the case of melanoma, while ruling out malignancy at an early stage may prevent unnecessary, costly, excisions and referrals to secondary care. Yet GPs in both the UK and the Netherlands receive only limited training in dermatology [13,14] and it has been suggested that GPs’ skills of diagnosing skin lesions could be improved [19]. On the other hand, one study found that melanoma are not likely to be excised more inadequately in primary care than they are in secondary care [20]. Nevertheless, ensuring that GPs and GP registrars acquire a satisfactory level of dermatological knowledge for the accurate diagnosis and treatment of skin cancer should have priority.

### Strengths and limitations

As in every healthcare database, the reliability is dependent on the accuracy of registration. Therefore all GPs participating in the RNG receive special training to maintain optimal registration. A limitation of this database is that it does not distinguish between consultations that were genuinely used for diagnosing skin lesions that were suspected of malignancy by either the patient or the GP and those that were not. However, we believe that, based on the selected ICPC codes, we predominantly identified suspected lesions. Because the reported numbers merely represent an indication of provided care rather than an absolute number of true skin lesions suspected of malignancy they should therefore be interpreted with caution. Also, in this database it is not clear whether the reason for the next consultation was prompted by exactly the same lesion or another lesion. As this may bias the percentage of patients subjected to minor surgery or who were referred to secondary care, we decided to consider only the data of the first visit and the following year for the analysis. However, we are confident that the analysis based on this large primary care database enabled us to draw valid conclusions on the burden that skin lesions suspected of malignancy impose on general practice. And although this study was conducted in the northern part of the Netherlands, we believe that with increasing incidence rates of skin cancer all over Europe [3-5], the observed trends in this study should be similar in other countries.

## Conclusions

Skin lesions that are suspected of malignancy impose an increasing burden on primary healthcare and most likely on healthcare costs as well. Especially, as many of these lesions are either excised or referred to secondary healthcare. General practitioners should therefore be trained in diagnosing these lesions, as a high diagnostic accuracy can save lives in the case of melanoma. Additionally, it may also prevent unnecessary, costly, excisions and referrals to secondary healthcare.

## Competing interests

The authors declare that they have no competing interests.

## Authors’ contributions

CK participated in the design of the study, analysis and interpretation of the data and drafted the manuscript. BK participated in the analysis and interpretation of the data and helped to draft the manuscript. FG participated in the acquisition and analysis of the data and helped to draft the manuscript. KvdM participated in the design of the study, interpretation of the data and helped to draft the manuscript. WvdH participated in the design of the study, interpretation of the data and helped to draft the manuscript. All authors read and approved the final manuscript.

## Pre-publication history

The pre-publication history for this paper can be accessed here:

http://www.biomedcentral.com/1471-2296/15/29/prepub

## Supplementary Material

Additional file 1 **Appendix.** ICPC codes. Click here for file

## References

[B1] Flohil SC de Vries E Neumann HA Coebergh JW Nijsten T Incidence, prevalence and future trends of primary basal cell carcinoma in the Netherlands Acta Derm Venereol 2011 91 1 24 30 21264452 10.2340/00015555-1009

[B2] Holterhues C Vries E Louwman MW Koljenovic S Nijsten T Incidence and trends of cutaneous malignancies in the Netherlands, 1989–2005 J Invest Dermatol 2010 130 7 1807 1812 10.1038/jid.2010.58 20336085

[B3] Wallingford SC Alston RD Birch JM Green AC Increases in invasive melanoma in England, 1979–2006, by anatomical site Br J Dermatol 2011 165 4 859 864 10.1111/j.1365-2133.2011.10434.x 21623751 PMC3407367

[B4] Lomas A Leonardi-Bee J Bath-Hextall F A systematic review of worldwide incidence of nonmelanoma skin cancer Br J Dermatol 2012 166 5 1069 1080 10.1111/j.1365-2133.2012.10830.x 22251204

[B5] Hollestein LM van den Akker SA Nijsten T Karim-Kos HE Coebergh JW de Vries E Trends of cutaneous melanoma in The Netherlands: increasing incidence rates among all Breslow thickness categories and rising mortality rates since 1989 Ann Oncol 2012 23 2 524 530 10.1093/annonc/mdr128 21543630

[B6] Hollestein LM de Vries E Nijsten T Trends of cutaneous squamous cell carcinoma in the Netherlands: increased incidence rates, but stable relative survival and mortality 1989–2008 Eur J Cancer 2012 48 13 2046 2053 10.1016/j.ejca.2012.01.003 22342554

[B7] de Vries E Nijsten T Louwman MW Coebergh JW Skin cancer epidemic in the Netherlands Ned Tijdschr Geneeskd 2009 153 A768 20025791

[B8] Know the 9 signals [http://scripts.kwfkankerbestrijding.nl/bestellingen/documents/Vroege%20ontdekking%20poster%20M.pdf]

[B9] Bosch MM Boon ME Malignant melanoma in a primary care pathologico-anatomical laboratory in 1988 and the freckle bus year Ned Tijdschr Geneeskd 1990 134 0028–2162; 42 2051 2054 2234179

[B10] Euromelanoma[http://www.euromelanoma.org/]

[B11] SunSmart[http://www.sunsmart.org.uk/]

[B12] de Vries E van de Poll-Franse LV Louwman WJ de Gruijl FR Coebergh JW Predictions of skin cancer incidence in the Netherlands up to 2015 Br J Dermatol 2005 152 0007–0963; 3 481 488 15787817 10.1111/j.1365-2133.2005.06386.x

[B13] Poelmann TA van der Heide WK Berendsen AJ Skin tumours underexposed in general practice Ned Tijdschr Geneeskd 2012 156 44 A5279 23114178

[B14] The National Institute for Health and Clinical Excellence (NICE) Improving Outcomes for People with Skin Tumours including Melanoma 2006 NICE: The Manual 31891468

[B15] Lamberts H Wood M The birth of the international classification of primary care (ICPC). Serendipity at the border of Lac leman Fam Pract 2002 19 0263–2136; 0263–2136; 5 433 435 12356688 10.1093/fampra/19.5.433

[B16] Lamberts H Wood M ICPC. International Classification of Primary Care 1987 Oxford: Oxford University Press

[B17] Cardol M van Dijk I de Jong JD de Bakker DH Westert GP Huisartsenzorg: wat doet de poortwachter? Tweede Nationale Studie naar ziekten en verrichtingen in de huisartspraktijk. Nivel/RIVM 2004 http://www.nivel.nl/sites/default/files/bestanden/ns2_rapport2.pdf

[B18] van Dijk CE Verheij RA Spreeuwenberg P Groenewegen PP de Bakker DH Minor surgery in general practice and effects on referrals to hospital care: observational study BMC Health Serv Res 2011 11 2 10.1186/1472-6963-11-2 21205305 PMC3024924

[B19] Pockney P Primrose J George S Jayatilleke N Leppard B Smith H Little P Kneebone R Lowy A Recognition of skin malignancy by general practitioners: observational study using data from a population-based randomised controlled trial Br J Cancer 2009 100 1 24 27 10.1038/sj.bjc.6604810 19127264 PMC2634694

[B20] Murchie P Sinclair E Lee AJ Primary excision of cutaneous melanoma: does the location of excision matter Br J Gen Pract 2011 61 583 131 134 10.3399/bjgp11X556272 21276340 PMC3026152

