# Supplementary material for: Skin lesions suspected of malignancy: an increasing burden on general practice
Source: BMC Fam Pract. 2014 Feb 12;15:29. doi: 10.1186/1471-2296-15-29 (PMC3930756; doi:10.1186/1471-2296-15-29)
Supplement: Additional file 1 — Appendix. ICPC codes. [file 1471-2296-15-29-S1.doc]

Appendix

ICPC codes

| code | Description |
| --- | --- |
| S26 | Fear of cancer of skin |
| S77 | Malignant neoplasm of skin |
| S79 | Benign neoplasm of skin, other |
| S80 | Unspecified neoplasm of skin, other |
| S81 | Haemangioma/lymphangioma |
| S82 | Naevus/mole |
| S83 | Congenital skin anomaly, other |
| S99 | Skin disease, other |

http://www.rivm.nl/who-fic/cdromthesaurus/Pagerenglish.pdf.
